# Anti-tumor effect and mechanistic study of elemene on pancreatic carcinoma

**DOI:** 10.1186/s12906-019-2544-2

**Published:** 2019-06-18

**Authors:** Jin Long, Zhe Liu, Lian Hui

**Affiliations:** 1 Department of General Surgery, The First Hospital of China Medical University, Shenyang, 110001 People’s Republic of China; 2 Department of Otolaryngology, The First Hospital of China Medical University, Shenyang, 110001 People’s Republic of China

**Keywords:** Elemene, Pancreatic carcinoma, Ectopic xenografts, Cell proliferation, Apoptosis, Cell cycle

## Abstract

**Background:**

Elemene is an effective anticancer component extracted from Zingiberaceae plants. This work was aimed to evaluate the anti-tumor effect and mechanism actions of elemene on pancreatic carcinoma in vitro and in vivo.

**Methods:**

The anti-proliferation experiment was measured by Methylthiazolyldiphenyl-tetrazolium bromide (MTT) method in the time of 24, 48 and 72 h in three different dosages. The cell cycle was detected by flow cytometer after 12 h treatment. Forty-eight nude mice were subcutaneously xenograft with BxPC-3 pancreatic cancer cells and divided into four groups: Control group and high, medium, low dosage of elemene (20, 40 and 60 mg/kg) treatment groups. Immunoblot and immunohistochemical methods were applied to detect the protein expression of P53 and Bcl-2 in the tumor of pancreatic cancer xenografts. H & E staining was used to detect the histopathological changes in each group.

**Results:**

A significant inhibition effect was observed in the anti-proliferation of BxPC-3 and Panc-1 cells in vitro in the time course of 24, 48 and 72 h with a dose dependent manner. The cell cycle results showed that elemene could arrest pancreatic cancer cells in the S phase after 12 h treatment in BxPC-3 and Panc-1 cell line. The in vivo BxPC-3 xenografts study exhibited that elemene was significantly decreased the tumor size in the high dosage group, compared to control group. And there is no any significant change in body weight of all animals. H&E pathology section result showed that treatment with elemene significantly decreased the inflammation cells and reduced the histopathological changes with a dose-dependent manner. Meanwhile, treatment with elemene significantly up-regulates the protein expression of P53, while down-regulate the protein expression of Bcl-2 in the tumor tissues, respectively. Furthermore, the western blot result showed that treatment with elemene increased the expression of P53 and decreased the expression of Bcl-2, compared with the control group, which is similar to the results of immunohistochemical staining.

**Conclusions:**

This study suggests that elemene has a potential anti pancreatic cancer effect, down-regulation the protein expression of Bcl-2 and up-regulation the protein expression of P53 in a dose dependent manner may be is the anti-tumor mechanism.

## Background

Elemene (Ele) is an effective anticancer component extracted from Zingiberaceae plants [1]. A large number of experimental study confirmed that elemene can promote tumor cell apoptosis and inhibit cell proliferation [2–4]. Currently, elemene is commonly used in clinic for tumor treatment in China, it is researched and developed by Dalian Institute of Medical Sciences (Dalian, China) and approved by FDA of China. The elemene oral emulsion or elemene injection used in the clinic mainly contains *β*-elemene as the main ingredient, and which also contains a small amount of *γ*-elemene and *δ*-elemene. Treatment of elemene has been widely used in clinical tumors, such as lung cancer, glioblastoma and prostate cancer, [5–9] but less used in pancreatic cancer, the related experimental study in this type cancer is very rare. So the mechanism of the occurrence and the development of pancreatic cancer remain to be further study.

Pancreatic carcinoma is one of the most frequent and malignant cancers in the world [10–12]. It is the 4th leading cause of cancer related death in Western countries, which with a less than 5% survival rate in 5 years [13–15]. Although there have some improvements in the surgical techniques and the adjuvant medical therapies, the mortality approaching the incidence have not changed in the past four decades [16, 17]. According to the latest global statistic data in 2012, 4.9 per 100,000 in men and 3.6 per 100,000 in women were incidence every year [18]. In the United States, the pancreatic cancer incidence and mortality is about 7.5 and 7.0%, respectively, and in 2015, there have about 48,960 people were to be diagnosed with pancreatic carcinoma and among them, there about 80% people (~ 40,560) would die from pancreatic carcinoma [19]. While in China, although there has a lower incidence of pancreatic cancer than United States, the incidence of pancreatic cancer in China has increased as fast as that worldwide recently. In 2010, there have 23,226 women and 34,509 men died from pancreatic tumor in China, which have exceeding that in the United States [20–22].

Based above all, it is emerging for developing and exploring the new drugs targeting against pancreatic cancer. So in the present study, we studied the antitumor effect and further try to explaining the mechanism actions of elemene in vitro with two pancreatic carcinoma cell lines (BxPC-3 and Panc-1) and in vivo using human tumor xenografts (xenograft BxPC-3 cell line) in immuno-deficiency nude mice. We evaluated the anti-proliferation effect after elemene treated cells in vitro. The tumor inhibition effect was evaluated in the xenograft nude mice with BxPC-3 cell line. The mechanisms of elemene in cell cycle and cell apoptosis were detected in tumor tissues in this study. Although there have some study reported the effect of elemene on pancreatic cancer, the work were not deep. In this study, we first combined the in vitro cellular effect and the in vivo xenografts effect to evaluate the efficacy of elemene with different concentrations.

## Methods

### Materials and reagents

Human pancreatic cancer cell line BxPC-3 and Panc-1 were obtained from ATCC (Manassas, VA, USA). Mouse anti human P53 monoclonal antibody was purchased from Cell signaling Technology (CST). Rabbit anti human Bcl-2 monoclonal antibody was purchased from Wuhan boster Biological Engineering Co., Ltd.; beta-actin mouse anti human monoclonal antibody was purchased from pocky Biotechnology (Suzhou) Co., Ltd..

Elemene injection (*β*-elemene) provided by Dalian Jingang Pharmaceutical Co. Ltd..

### Cell culture

BxPC-3 and Panc-1 cells were cultured with DMEM containing 10% fetal bovine serum and 1% penicillin-streptomycin culture medium. The cells cultured in the humidification incubator at 37 °C and 5% CO_2_. Every 2–3 days in culture medium and passage. The logarithmic phase cells were digested with 0.25% trypsin and cells count collection and centrifugation, the concentration of the cells was adjusted to 1 × 10^7^/mL by vaccination for use.

### Anti-proliferation activity assay

The anti-proliferation activity of Ele in two pancreatic cancer cell lines (BxPC-3 and Panc-1) was measured by MTT (3-(4,5-dimethyl-2-thiazolyl)-2,5-diphenyl-2-H- tetrazolium bromide) method. The logarithmic growth phase cells of BxPC-3 and Panc-1 were planted in a 96-well plates with a concentration of 4 × 10^4^/mL, and 200 μL per well, respectively. The cells were incubated at the cell culture incubator at 37 °C for 12 h before the drug added. Then different concentrations of drug and vehicle solution (DMSO) were diluted with cell culture media (the stock drug concentration is 1000×), and replaced the medium into 96 well plate. After add the drug, the cells continue incubated at 37 °C for 24 h, 48 h and 72 h respectively. Add 20 μL of MTT solution to each well in the indicated times and incubate for 4 h. After MTT incubation, the cell plate were centrifuged at 1000 rpm, 10 min to make sure all the formazan crystals are adhered in the bottom of the flat plate, then discard the MTT supernatants carefully, finally add 100 μL DMSO to each well, shake plates for 10 min on a plateshaker by slowly increasing the shaking speed to a maximum of 900 shakes/min, to make sure the formazan crystals were thoroughly solved, then the absorbance of the plate were measured at a wavelength of 570 nm by a microplate reader. The cell viability was calculated with the formula: cell viability % = Absorbance value of drug/Absorbance value of vehicle × 100. Each test concentration was performed in triplicates and repeated three times.

### Cell cycle analysis

After treated for 12 h, the cells of BxPC-3 and Panc-1 were fixed with 70% ethanol at 4 °C for 12 h, and then washed with 1 × PBS to decant the ethanol solution. Then the cells were suspended in 100 μg/mL propidium iodide staining solution, and incubated in the dark at room temperature for 30 min. The cells were analyzed using a FACS Calibur (Becton, Dickinson and Company, USA). The distribution of cells in the various cell-cycle phases was analyzed with the CellQuest Pro software (Becton, Dickinson and Company, USA). All experiments were performed in triplicate.

### Animals

Forty-eight female BALB/C nude mice (body weight 20 ± 2 g) were provided by the animal experimental center of the China Medical University (Shenyang, China). Six mice were raised in one polyacrylic cage, and all the mice were quarantined for 1 week before the use. All the mice were reared in the animal experimental center of China Medical University in SPF grade environment with free access to food and water (24 ± 1 °C, 50% ± 5% of humidity and 12 h day/night cycle). The mice were received humane care in the terms of National Institutes of Health Guidelines of the USA (National Research Council of USA, 1996) and the University ethical regulations of China Medical University.

### Experimental design

The human pancreatic cancer cell line BxPC-3 was inoculated into the left axilla in BALB/C nude mice (1 × 10^6^/mice). When tumor volume was about 100 mm^3^ after tenth days of inoculation, the nude mice were randomly divided into four groups (Tumor control group, Ele 20 mg/kg group, Ele 40 mg/kg group and Ele 60 mg/kg group), and 12 mice in each group. Control group were injected with Intralipid 10 mL/kg/day body weight (BW) by intraperitoneal injection; Elemene groups were injected with elemene at 20, 40 and 60 mg/kg/day by 10 mL/kg BW through intraperitoneal injection, respectively.

At the end of the experiment, all the mice were sacrificed by cervical dislocation (all the mice were fasted about 12 h before harvest the samples), which about 28 days after inoculation. The tumor tissues were harvested, weighted and divided into two parts of each tumor tissue: one part fast refrigerated by liquid nitrogen and stored at − 80 °C for Western blot detection. The other part were fixed in the 40 g/L Formaldehyde Solution, and embedded into paraffin for immunohistochemical analysis.

### Western blot

The protein expression of P53 and Bcl-2 in the tumor tissues in each group were detected by Western blot. The pre-cold frozen tissues were washed with the pre-cold PBS buffer and put it into the pre-cold tissue homogenizer and added homogenate buffer (50 mmol/L, pH 7.5 Tis-HCl, 150 mmol/L NaCl, 1 mmol/L phenyl methyl sulfonyl fluoride, 1 mg/mL aprotinin, 4 mg/mL leupeptin), homogenized in ice bath, centrifugation at 4 °C, 10000 rpm/min and then collected the supernatant. The protein concentration was measured by BCA method. The samples loaded at 10% SDS-PAGE electrophoresis gel (50 μg protein/hole) for running. The protein bands were transferred from gel to PVDF membrane at 4 °C, 100 V for 1.5 h, and then the PVDF membrane was blocked under the 5% non-fat milk at room temperature for 1 h. The first antibody were (P53, 1:1000; Bcl-2, 1:500; beta-actin, 1:10000) incubated at 4 °C for overnight and the second antibody (invitrogen, 1:5000) were incubated at room temperature for 1 h. Finally, the membrane was incubated at ECL (Enhanced chemiluminescent substrate for horseradish peroxidase (HRP), Thermo Fisher Scientific) reagent for 1 min, and the image were scanned at Bio-Rad imager (Bio-Rad ChemiDoc MP, USA), the quantification values of the protein bands were analyzed by the Bio-Rad Image Lab Software 5.2.1 (Bio-Rad, USA).

### Histopathological analysis

Tumor specimens were fixed in the 40 g/L Formaldehyde Solution overnight. The fixed specimens were embedded in paraffin, cut into 5 μm thickness sections and then stained with hematoxylin-eosin (H&E) in terms of the routine histopathological examination. The final stained sections were photographed under a light microscope (BX-50 Olympus) at 200 × magnification.

### Immunohistochemistry assay

The protein expression of P53 and Bcl-2 in tumor tissues was detected by immunohistochemistry method in each group. The immunohistochemical method was used by EnVision two step methods, kit was purchased from Beijing Zhongshan biotechnology company (Beijing). 5 μm thickness of paraffin-embedded sections were mounted on glass slides, deparaffinized and quenched the endogenous peroxidase activity in 3% H_2_O_2_ for 10 min. Then the sections were incubated with the primary antibody overnight for the mouse anti P53 antibody and mouse anti Bcl-2 antibody at 4 °C, respectively. In the following, the sections were blocked by normal goat serum for 20 min. Next, the sections were incubated with the second HRP-conjugated goat anti-mouse antibody 30 min at 37 °C, respectively. Finally, the positive immunoreactive staining was visualized by incubated with DAB-H_2_O_2_ for 10 min at room temperature. Images were obtained at 200 × magnification, compared to the original image (Olympus BX-50 Microscope and a Leica DMI; Leica Microsystems).

### Statistical analysis

Values were represented as mean ± SD. All statistical comparisons were calculated by means of a one-way ANOVA test followed by Dunett’s t-test with SPSS19.0 statistical software. *P* < 0.05 and < 0.01 were regarded as statistically significant.

## Results

### Anti-proliferation activity of Ele on BxPC-3 and Panc-1 cell line

In order to test the anti-tumor effect of Ele, we first tested the anti-proliferation activity of Ele in two pancreatic cancer cell lines (BxPC-3 and Panc-1) in vitro. As showed in Table 1**,** the cell viability of BxPC-3 and Panc-1 cell lines were significantly decreased in Ele individual treatment dosage of 15, 30 and 60 μg/mL (*P* < 0.05, *P* < 0.01) with a dose dependent manner, compared with the control group. While followed with the treated times in 24, 48 and 72 h, the cell viability values were also significantly decreased, especially in BxPC-3 cells, which showed only 15.3% cells were survived. In addition, from the data of the anti-proliferation activity in vitro, we can see that BxPC-3 pancreatic cancer cell line was more sensitive to Panc-1 pancreatic cancer cell line (Table 1).

**Table 1:** Anti-proliferation activity for elemene on BxPC-3 and Panc-1 cell line

Group	Dosage (μg/mL)	CV% (BxPC-3)			CV% (Panc-1)		
		24 h	48 h	72 h	24 h	48 h	72 h
Control	–	100.0	100.0	100.0	100.0	100.0	100.0
Elemene	15	87.2 ± 3.2*	74.2 ± 3.0*	65.7 ± 2.8*	93.6 ± 3.3*	81.1 ± 3.6*	66.5 ± 2.7*
	30	57.7 ± 2.1**	46.1 ± 2.4**	41.7 ± 1.7**	70.5 ± 2.5**	59.3 ± 2.4**	47.6 ± 1.8**
	60	34.6 ± 2.0**	22.7 ± 1.5**	15.3 ± 0.9**	54.7 ± 1.3**	38.9 ± 0.8**	26.8 ± 0.9**

Data are expressed as mean ± SD for each group. *P < 0.05, **P < 0.01 vs control group. *CV* cell viability

### The cell cycle distribution of BxPC-3 and Panc-1 cell line after Ele treatment

Based on the result of in vitro anti-proliferation activity, next we would like to know the deeply effect of Ele, how was the cell cycle distribution like treated by Ele. From Table 2**,** it was showed that Ele could arrest the pancreatic cancer cells in S phase, which also could block the G2/M phase (*P* < 0.05, *P* < 0.01). This phenomenon was significantly increased in the higher dosage of Ele in 12 h (*P* < 0.05, *P* < 0.01). Furthermore, the cell cycle distribution result was consistent with the in vitro anti-proliferation activity, which also showed a sensitivity effect in BxPC-3 pancreatic cancer cell line (Table 2).

**Table 2:** The cell cycle distribution on BxPC-3 and Panc-1 cell line after 12 h of elemene treatment

Group	Dosage (μg/mL)	BxPC-3			Panc-1		
		G0/G1	S	G2/M	G0/G1	S	G2/M
Control	–	75.8 ± 1.9	18.6 ± 0.8	5.6 ± 0.05	86.1 ± 1.2	6.3 ± 0.07	7.6 ± 0.04
Elemene	15	61.2 ± 1.3*	34.6 ± 0.7*	4.2 ± 0.03*	78.3 ± 0.9*	15.2 ± 0.6*	6.5 ± 0.03*
	30	47.7 ± 0.9**	49.1 ± 0.6**	3.2 ± 0.02**	60.1 ± 0.7**	34.8 ± 0.9**	5.1 ± 0.02**
	60	30.6 ± 0.5**	66.7 ± 0.8**	2.7 ± 0.09**	46.3 ± 0.8**	50.1 ± 1.1**	3.6 ± 0.04**

Data are expressed as mean ± SD for each group. *P < 0.05, **P < 0.01 vs control group

### Anti tumor effect of Ele in BxPC-3 ectopic xenografts

**Fig. 1:**
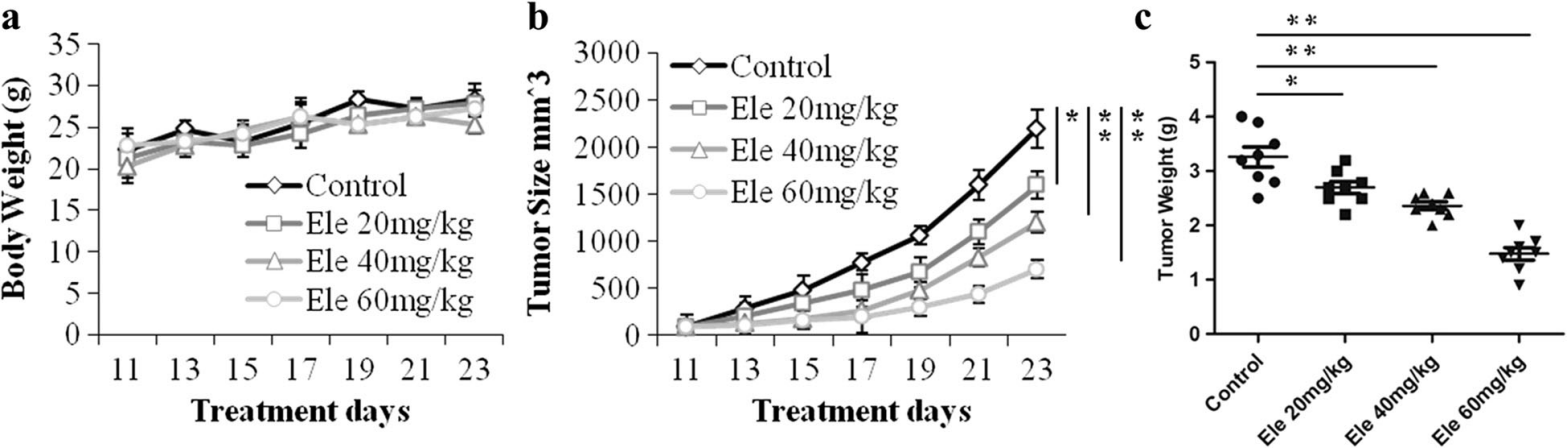
Effects of elemene on body weight (**a**), tumor size (**b**) and tumor weight (**c**). ^*^P < 0.05, ^**^P < 0.01 vs control group. Ele: elemene

Under the result of the in vitro, we continue studied the anti-tumor effect of Ele in BxPC-3 ectopic xenografts through subcutaneous transplantation. As Fig. 1 showed, Ele has a remarkable anti-tumor effect in BxPC-3 ectopic xenografts in vivo (*P* < 0.05, *P* < 0.01), compared with the control group. After continues 14 days Ele treatment, the tumor size were significantly reduced in the dosage of 20, 40 and 60 mg/kg with a dose dependent manner (Fig. 1b, *P* < 0.05, *P* < 0.01). The anti-tumor effect in vivo was also reflected from the statistic data of tumor weight in three different dosage of Ele treatment (Fig. 1c, *P* < 0.05, *P* < 0.01). In addition, from the whole in vivo anti-tumor experiment, all the animals are alive, and the statistic data of the animal body weight showed that treatment with Ele did not influence the animal body weight, even in the high dosage of 60 mg/kg (Fig. 1a), which demonstrated that Ele has a lower toxicity in vivo.

### The protein expression of P53 and Bcl-2 in tumor lysate by western blot

**Fig. 2:**
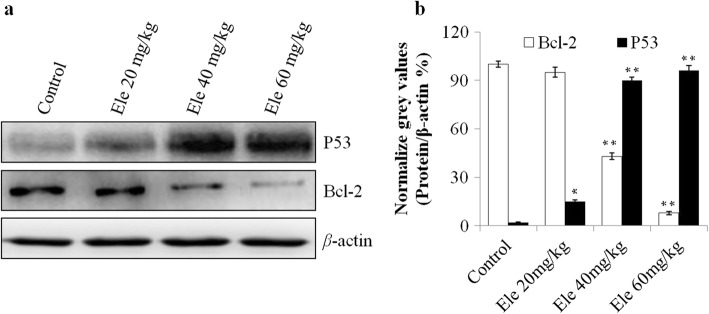
**a** Representative photographs of P53 and Bcl-2 expression on xenograft tumor lysate. **b**: Normalize grey values of P53 and Bcl-2 expression. ^*^P < 0.05, ^**^P < 0.01 vs control group

In the in vivo anti-tumor experiment, we also detected the expression of P53 and Bcl-2 in the different groups by Western blot method (Fig. 2). The protein expression of P53 can be detected in the protein bands of 53 kDa and Bcl-2 can be detected in the protein bands of 26 kDa, and in this experiment, we used *β*-actin as the inner protein reference, which can be detected in 43 kDa (Fig. 2). From Fig. 2, the statistical analysis showed that the protein expression of P53 in each treatment groups were significantly increased (*P* < 0.05, *P* < 0.01) with a dose dependent manner, compared to the negative control group. While the protein expression of Bcl-2 were showed statistically significant differences between each groups, the Ele treatment groups were significantly decreased the protein expression of Bcl-2 (*P* < 0.05, *P* < 0.01), especially in the dosage of 60 mg/kg (Fig. 2b, *P* < 0.01). This result showed that the anti-tumor effects of Ele maybe influenced the tumor suppressor gene and the apoptosis related gene.

### Histopathological changes in the liver tissue

**Fig. 3:**
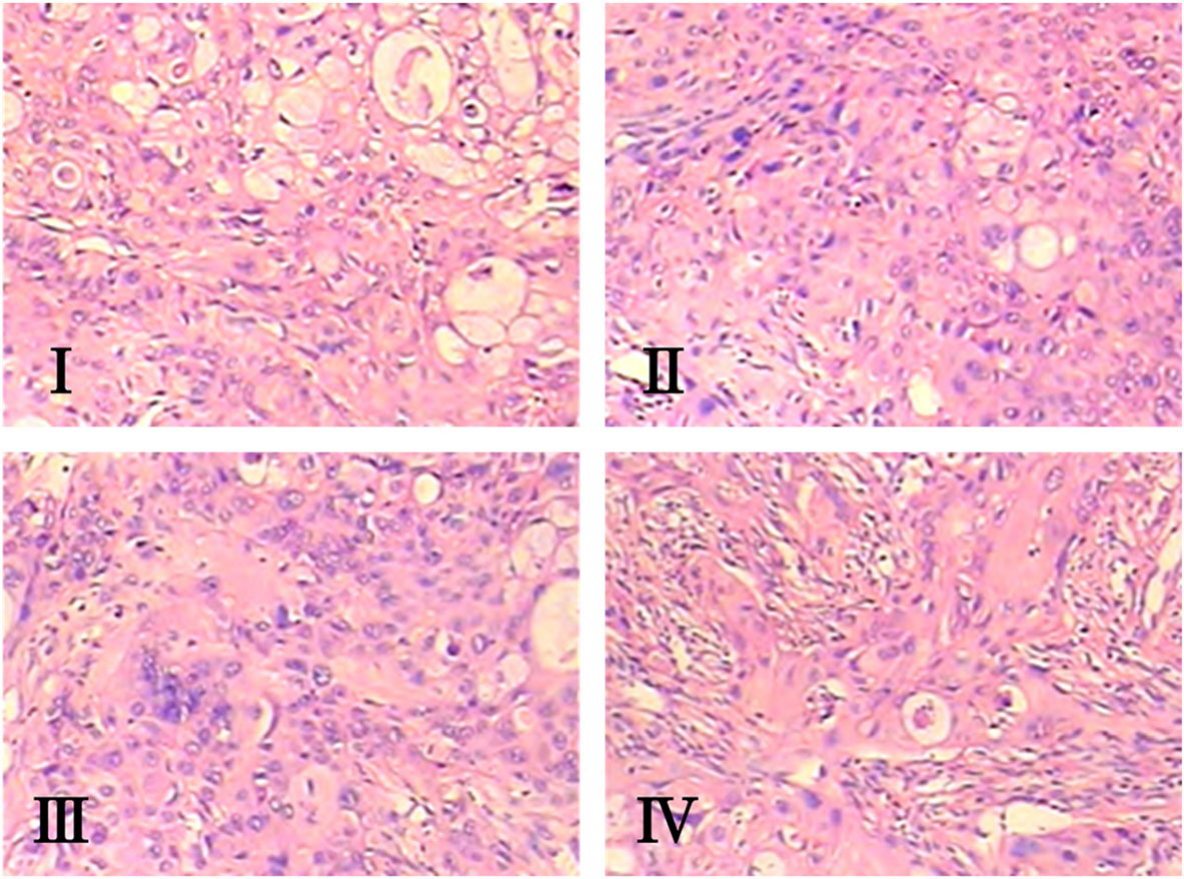
Histopathological examinations by H&E (B, 200 ×). I: Control group, II: Elemene 20 mg/kg group, III: Elemene 40 mg/kg group, IV: Elemene 60 mg/kg group

For the changes of transplantation tumor histology, we conducted H&E routine staining for each transplanted tumor tissues. Under the photomicroscope, the negative tumor control group was observed part of gland cancer nests, part of a tubular part, intracavitary mucus retention, not a uniformitarian nuclear size, abnormal cancer tissues and peripheral lymphocyte infiltration (Fig. 3 I). Meanwhile the tumor sections from the low dose group (20 mg/kg) showed that there have a less adenocarcinoma glandular cavity mucus, more visible lymphocyte infiltration in cancer and peripheral tissue (Fig. 3 II). While the medium dose group (40 mg/kg) showed more acute inflammatory cell infiltration in cancer tissue and peripheral tissue (Fig. 3 III), and the high dose group (60 mg/kg) showed degeneration and necrosis of cancer tissue, decreased secretory cells and a large number of acute and chronic inflammatory cell infiltration in cancer tissue and peripheral tissue (Fig. 3 IV).

### Effect of Ele on IHC analysis of tumor P53

**Fig. 4:**
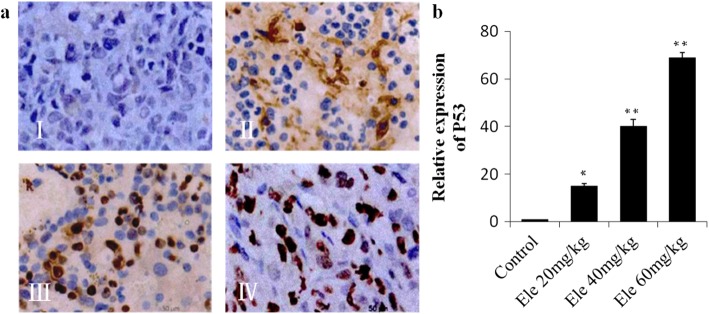
**a** Representative photographs of P53 immunological histological chemistry examination (200 ×). I: Control group, II: Elemene 20 mg/kg group, III: Elemene 40 mg/kg group, IV: Elemene 60 mg/kg group. **b** Quantification of P53 stained cells. ^*^P < 0.05, ^**^P < 0.01 vs control group

Immunohistochemistry was used to detect the expression of P53 in transplanted tumor. The positive P53 expression was showed as brown granules in human pancreatic cancer xenografts in nude mice, which mainly distributed in the nucleus (Fig. 4). The positive nucleus expression of P53 in the negative tumor control group was less (Fig. 4 I), while in the Ele treatment group, the positive nucleus P53 expression was significantly increased with a dose dependent manner (Fig. 4 II-IV, *P* < 0.05, *P* < 0.01).

### Effect of Ele on IHC analysis of tumor Bcl-2

**Fig. 5:**
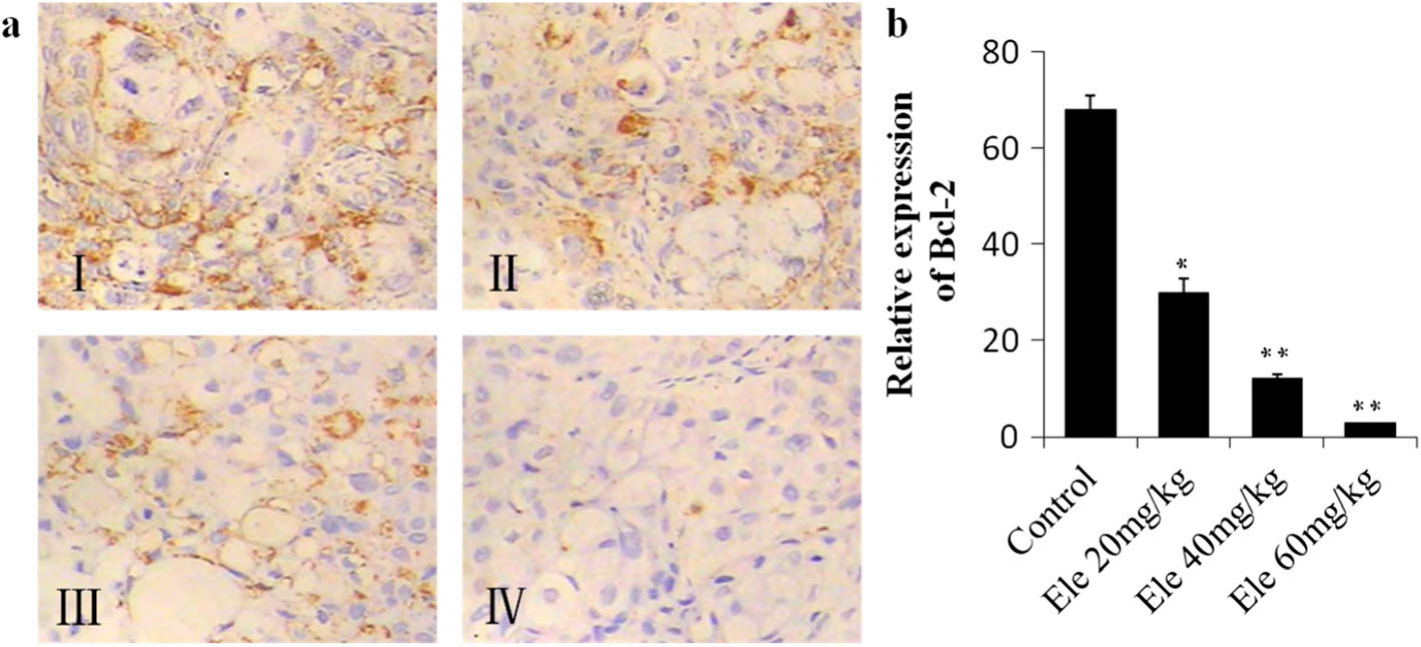
**a** Representative photographs of Bcl-2 immunological histological chemistry examination (200 ×). I: Control group, II: Elemene 20 mg/kg group, III: Elemene 40 mg/kg group, IV: Elemene 60 mg/kg group. **b** Quantification of P53 stained cells. ^*^P < 0.05, ^**^P < 0.01 vs control group

Figure 5 showed the result of the expression of Bcl-2 in transplanted tumor tissues by immunohistochemistry analysis. The positive Bcl-2 expression showed as brown granules in human pancreatic cancer xenografts in nude mice, which mainly distributed in the cytoplasm (Fig. 5). There have more positive Bcl-2 expression in the negative control group (Fig. 5 I), while the positive Bcl-2 expression were significantly decreased in the Ele treatment groups, especially the high doses 60 mg/kg group (Fig. 5 IV, *P* < 0.01). Figure 5b showed the statistically data of the quantification of positive P53 stained cells.

## Discussion

Pancreatic carcinoma is one of the malignant tumors in digestive system in the USA. It is the fourth leading cause of the cancer-related death and the 5 years survival rate is about 6% [23, 24]. While in all the newly discovered cancer patients in the world, there are more than 2% cancer patients are pancreatic cancer patients [25]. In China, the numbers of the pancreatic cancer patient was increased year by year, and the trend of the patients’ age was younger and younger. Although the radiotherapy, chemotherapy and other therapy means have certain effect to prolong the survival period of the patient, the overall survival is still less than 24 month [26]. In addition, there has a certain resistance to conventional chemotherapy (eg. gemcitabine) for pancreatic cancer [27, 28]. Therefore, there is an urgent need to find an effective drug treatment and treatment strategies. For in vivo anti-tumor effect evaluation, the xenografts of the mice are one of the important models. While the immunodeficiency mice often used as the tumor growth vector. In this study, we used nude mice and constructed the human pancreatic cancer xenografts in nude mice and studied the related treatment mechanism of elemene in vivo and in vitro.

The anti-tumor proliferation activity of drugs is the basic index for anti-tumor effect in vitro. Since all the tumor cells have a common feature that is growth faster, thus, determine the anti-proliferation of the drugs is one of the important factors for its anti-tumor effects. In addition, block the cell cycle of the tumors also is an important factor for drug’s anti-tumor effect. In this study, we found that treatment with Ele significantly decreased the cell viability of BxPC-3 and Panc-1 cells. While in the cell cycle test, it was showed that Ele could arrest the pancreatic cancer cells in S phase, which also could block the cells in G2/M phase. This phenomenon reflected that Ele have an anti-tumor effect in vitro.

The Bcl-2 family protein is an important regulatory molecule involved in the regulation of cell apoptosis. It is divided into two categories, one is the anti apoptotic proteins, such as Bcl-2, and the other is a pro apoptotic protein, such as Bax. The sensitivity of cells to apoptotic depends on the ratio of anti apoptotic proteins and pro apoptotic protein [29, 30]. The P53 protein encoded by tumor suppressor P53 gene, which regulated the cell apoptosis through the P53-Bax mitochondrial pathway [30–32]. Some research reported that elemene could induce cell apoptosis through the mitochondria mediated caspase pathway. In addition, some research also reported that beta elemene can reduce the expression of Bcl-2 in lung cancer and prostate cancer, increase the expression of cytochrome C, ADP ribose polymerase (PARP), caspase 3, caspase7 and caspase 9 [33] with a dose dependent manner in in vivo anticancer effect, and the half antitumor inhibitory concentration values (50% Inhibiting concentration, IC_50_) are up to hundreds of micromole in different types of tumor cells [34–36].

In the present research work, the results showed that treatment with elemene significantly decreased the tumor size and tumor weight in all dosage of elemene. While in the high dosage (60 mg/kg) treatment group of pancreatic cancer, elemene was markedly increased the protein expression of P53 and decreased Bcl-2 protein expression (*P* < 0.01). Furthermore, there also displayed the increase protein expression of P53 and the decrease Bcl-2 protein expression in the low dosage group (20 mg/kg, *P* < 0.05), but not as good as the high dose group, which suggesting that the regulation of pancreatic cancer on the protein levels of P53 and Bcl-2 with a dose dependent relationship. Therefore, based on the above results, the anti-tumor mechanism of elemene maybe related to promote the apoptosis of pancreatic carcinoma cells, which is consistent with other elemene research results in cancers.

## Conclusion

This study demonstrated that elemene has an antitumor effect in pancreatic carcinoma in vivo and in vitro. It can inhibit the proliferation of pancreatic cell lines in vitro, and which mainly arrest the cells to S phase and also block the G2/M phase in the cell cycle analysis. The in vivo antitumor study showed that elemene could significantly decrease the tumor size in BxPC-3 xenografts in nude mice, but not decrease the body weight of the experiment mice, which exhibited that elemene has a lower toxicity and more safe to be use in vivo. Therefore, since the lower cytotoxicity of elemene, it is expected to become a new chemotherapy drugs in clinical treatment of pancreatic cancer. Of course, the deeply mechanism of elemene against pancreatic carcinoma and its clinical application remains to be further research and evaluation.

## Data Availability

The datasets used and/or analysed during the current study are available from the corresponding author on reasonable request.

## Abbreviations

BW: Body weight
Ele: Elemene
H&E: Hematoxylin and eosin
